# Advancements in AI Medical Education: Assessing ChatGPT’s Performance on USMLE-Style Questions Across Topics and Difficulty Levels

**DOI:** 10.7759/cureus.76309

**Published:** 2024-12-24

**Authors:** Parker Penny, Riley Bane, Valerie Riddle

**Affiliations:** 1 Medical Education, University of South Florida Morsani College of Medicine, Tampa, USA; 2 Medical Education, College of Arts and Sciences, Florida State University, Tallahassee, USA

**Keywords:** ai, artificial intelligence, chat-gpt, medical education, usmle

## Abstract

Background

AI language models have been shown to achieve a passing score on certain imageless diagnostic tests of the USMLE. However, they have failed certain specialty-specific examinations. This suggests there may be a difference in AI ability by medical topic or question difficulty. This study evaluates the performance of two versions of ChatGPT, a popular language-based AI model, on USMLE-style questions across various medical topics.

Methods

A total of 900 USMLE-style multiple-choice questions were equally divided into 18 topics, categorized by exam type (step 1 vs. step 2), and copied from AMBOSS, a medical learning resource with large question banks. Questions that contained images, charts, and tables were excluded due to current AI capabilities. The questions were entered into ChatGPT-3.5 (version September 25, 2023) and ChatGPT-4 (version April 2023) for multiple trials, and performance data were recorded. The two AI models were compared against human test takers (AMBOSS users) by medical topic and question difficulty.

Results

Chat-GPT-4, AMBOSS users, and Chat-GPT-3.5 had accuracies of 71.33%, 54.38%, and 46.23% respectively. When comparing models, GPT-4 was a significant improvement demonstrating a 25% greater accuracy and 8% higher concordance between trials than GPT-3 (p<.001). The performance of GPT models was similar between step 1 and step 2 content. Both GPT-3.5 and GPT-4 varied performance by medical topic (p=.027, p=.002). However, there was no clear pattern of variation. Performance for both GPT models and AMBOSS users declined as question difficulty increased (p<.001). However, the decline in accuracy was less pronounced for GPT-4. The accuracy of the GPT models showed less variability with question difficulty compared to AMBOSS users, with the average drop in accuracy from the easiest to hardest questions being 45% and 62%, respectively.

Discussion

ChatGPT-4 shows significant improvement over its predecessor, ChatGPT-3.5, in the medical education setting. It is the first ChatGPT model to surpass human performance on modified AMBOSS USMLE tests. While there was variation in performance by medical topic for both models, there was no clear pattern of discrepancy. ChatGPT-4’s improved accuracy, concordance, performance on difficult questions, and consistency across topics are promising for its reliability and utility for medical learners.

Conclusion

ChatGPT-4's improvements highlight its potential as a valuable tool in medical education, surpassing human performance in some areas. The lack of a clear performance pattern by medical topic suggests that variability is more related to question complexity than specific knowledge gaps.

## Introduction

AI language models are being integrated into medical education at a rapid rate. The American Medical Association highlights how institutions like NYU Grossman School of Medicine are using these tools to enhance precision education, enabling educators to provide personalized learning experiences tailored to individual student needs [1]. Other literature on the topic has suggested that AI could be used as a virtual teacher’s assistant for medical students, facilitating interactive simulations, or supplementing exam preparation [2-4]. There has even been investigation into using language models to write medical exams, saving time, money, and relieving the burden on faculty [4,5]. However, even with AI’s apparent promise, the accuracy and knowledge base of these models relating to medical school education remain in question. One approach to assessing the knowledge base of AI as a medical education tool has involved administering standardized medical exams to these models. Chat-GPT, a language-based AI, was the first of its kind to achieve the benchmark of performing at the passing threshold of the USMLE, a set of multiple-choice, standardized tests required to demonstrate competency for medical licensure in the United States [6]. It has also proved to be capable of certain specialized, medical specialty-specific examinations such as those in neurosurgery and ophthalmology [7-10]. However, Chat-GPT has been shown to perform poorly and fall short of passing on other specialty-specific examinations such as the American College of Gastroenterology self-assessment tests [11]. While ChatGPT-4 has shown enhanced medical knowledge compared to the earlier GPT-3.5 model, the variation in AI performance among medical assessments suggests that AI capabilities may differ by medical topic [12,13]. It has been proposed that AI may struggle with theoretical concepts in more cerebral fields such as cardiology or neurology and that it may do better with medical knowledge rather than medical soft skills, such as navigating interpersonal scenarios and exercising ethical judgments [14-16].

USMLE-style questions have been previously used to evaluate ChatGPT's capabilities; however, analyses have yet to assess variability in performance across all medical topics [17]. We investigated Chat-GPT's performance on USMLE-style questions, hypothesizing there would be a difference in ability by medical topic. We also compared the performance of GPT-3.5 and GPT-4 to that of human test takers.

## Materials and methods

Question selection

This study accessed 900 USMLE-style multiple-choice questions from AMBOSS, a popular medical learning resource with a large question bank (Table 2 of Appendices) [18]. Questions were selected based on current AI capabilities where those that contained images, charts, and tables were excluded. The questions were separated by exam (step 1 vs. step 2) and divided among 18 topics relevant to medical education, such that each topic for each exam had a sample of 25 questions. Each question had a reported difficulty represented as a categorical range from one to five based on AMBOSS user historical performance. The questions also had a reported average percent correct score provided by AMBOSS, which includes data only from users who answered the question on their first attempt and took at least five seconds to choose their answer. The 18 topics were as follows: Behavioral Health, Biostatistics/Epidemiology/Population Health and Interpretation of Medical Literature, Blood and Lymphoreticular Systems, Cardiovascular System, Endocrine System, Female Reproductive System and Breast, Gastrointestinal System, General Principles of Foundational Science, Immune System, Male Reproductive System, Multisystem Processes and Disorders, Musculoskeletal System, Nervous System and Special Senses, Pregnancy and Childbirth and the Puerperium, Renal and Urinary Systems, Respiratory System, Skin and Subcutaneous Tissue, and Social Sciences.

Data collection

The questions were sourced directly from AMBOSS and entered verbatim into the online versions of ChatGPT. This included the question and the multiple-choice answers labeled alphabetically, with care to make sure there were no formatting inconsistencies that could help the AI model reach the correct answer. Each sample of 25 multiple-choice questions was input into both ChatGPT-3.5 (version September 2023) and ChatGPT-4 (version April 2023) separately. Two trials were conducted for each question set using each AI version, utilizing different GPT input interfaces for each trial. This resulted in a total of four trials for each GPT model. A binary correctness score was given for each question in each trial, and a percent correct score was calculated for each sample set. The results were compared between trials for concordance and averaged to account for variation. The results between step exam content were also averaged. The performance of the AI models was compared to AMBOSS users and analyzed by medical topic, difficulty, and USMLE exam.

Statistical analysis

Data were analyzed using SPSS statistical software (IBM Corp., Armonk, NY). ANOVA was used to evaluate overall performance differences among GPT-4, GPT-3.5, and AMBOSS users, as well as performance across different subject areas and question difficulties. Post-hoc Tukey’s tests were conducted to examine specific pairwise comparisons. Additionally, we analyzed concordance between trials and assessed changes in performance across question difficulty levels. We also assessed performance between step exams.

## Results

Table 1 shows the overall performance of the GPT models and the AMBOSS users. ChatGPT-4 performed significantly better overall than AMBOSS users, which performed better than ChatGPT-3.5 (p<.001). GPT-4 also had superior concordance between trials than GPT-3.5 (p<.001). Performance was similar between step 1 and step 2 content for both models.

**Table 1:** Descriptives of overall Chat-GPT and AMBOSS user performance This data depicts percent correctness scores and is represented as mean (95% confidence interval upper limit - lower limit).

	Step 1 (n=450)	Step 2 (n=450)	Combined (n=900)
GPT-3.5 % correct	46.67 (42.54-50.79)	45.78 (41.63-49.91)	46.22 (43.31-49.13)
GPT-4 % correct	71.44 (67.62-75.27)	71.22 (67.24-75.07)	71.33 (68.60-74.06)
AMBOSS Users % correct	52.89 (51.14-54.64)	55.77 (53.96-57.59)	54.38 (53.11-55.64)
GPT-3.5 concordance %	79.56 (75.82-83.30)	80.20 (76.48-83.88)	79.78 (77.15-82.41)
GPT-4 concordance %	86.47 (83.27-89.62)	89.14 (86.19-91.98)	87.78 (85.63-89.92)

**Figure 1:**
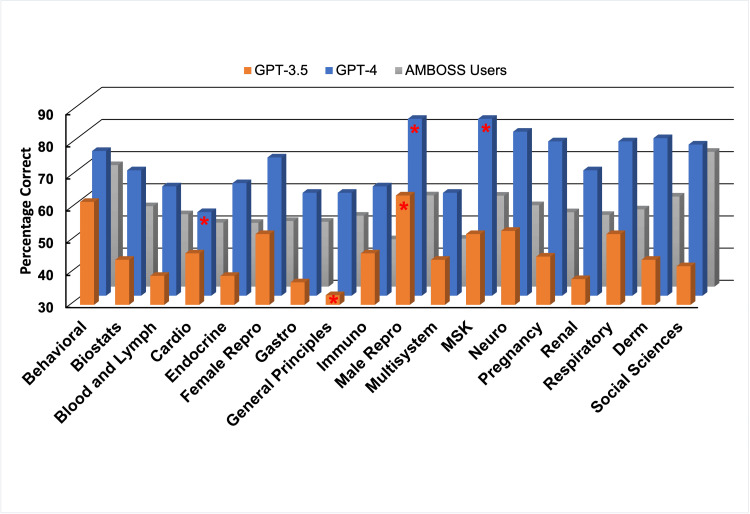
Performance of Chat-GPT and AMBOSS users by topic Statistical significance was determined using ANOVA with post-hoc Tukey’s tests at a p-value <0.05.

Figure 1 shows performance by medical topic. Both GPT-3.5 and GPT-4 had varied performance by topic (p=.027, p=.002). GPT-3.5 had the lowest topic accuracy in general principles of foundational science at 33.0%. GPT-4 had the lowest topic accuracy in the cardiovascular system at 56.0%. Both models had the highest accuracy with the male reproductive system at 64.0% and 85.0%, respectively. GPT-4 also had 85.0% accuracy with the musculoskeletal system. Concordance changed by section for GPT-4 but not GPT-3.5 (p=.001, p=.118). 

**Figure 2:**
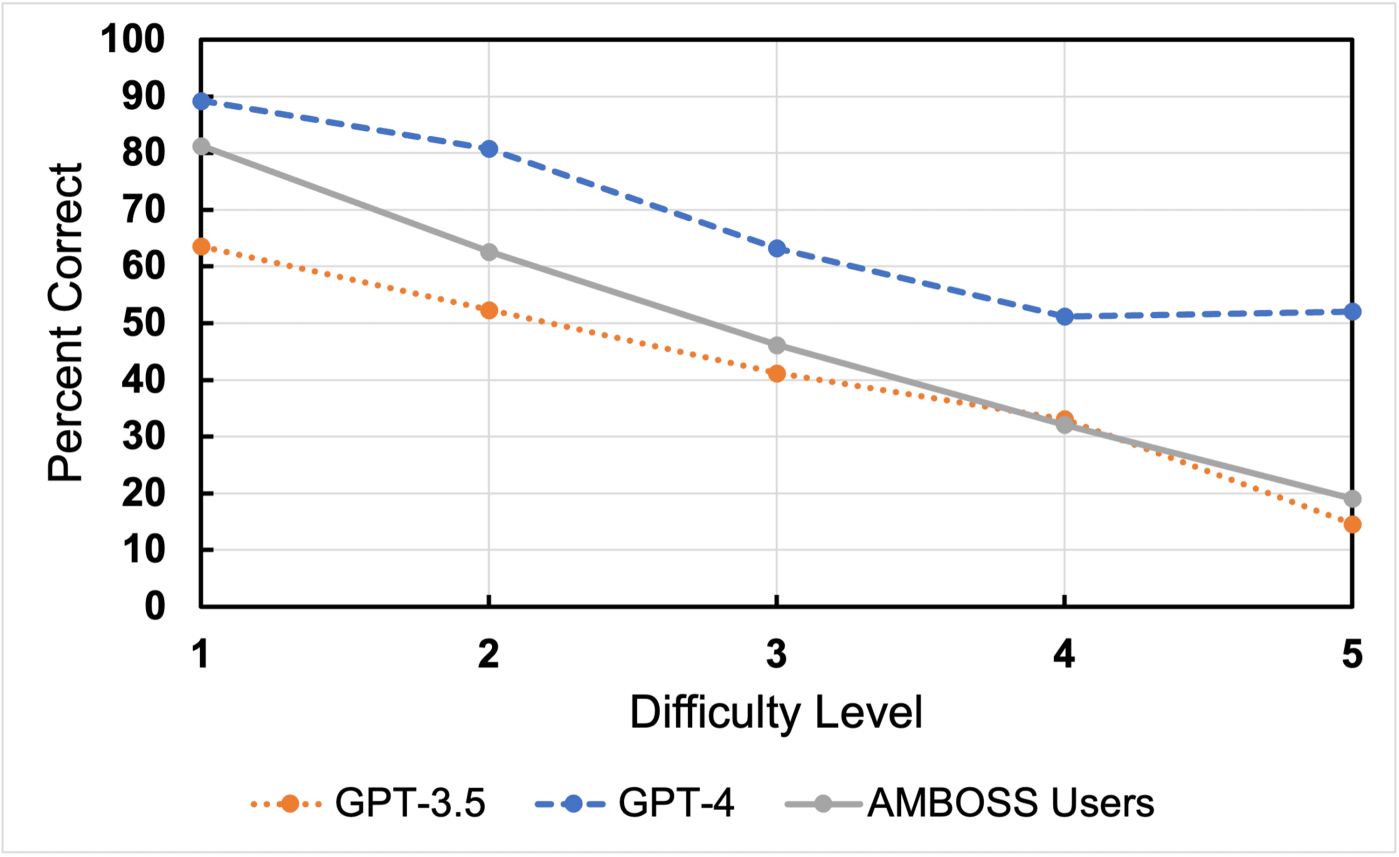
Performance of Chat-GPT and AMBOSS users by question difficulty This data depicts percent correctness scores and is plotted by question difficulty level with trend lines drawn between each data point.

Figure 2 shows performance by question difficulty. Performance for both GPT models and AMBOSS users declined as question difficulty increased (p<.001). However, the decline in accuracy was less pronounced for GPT-4. From the easiest to hardest level of questions, GPT-4, GPT-3.5, and AMBOSS users had a drop in accuracy of 37.6%, 48.4%, and 61.9%, respectively. Concordance changed by difficulty for GPT-4 where higher difficulty questions had lower concordance (p<.001). Concordance did not change with question difficulty for GPT-3.5 (p=.748). 

There was significant variance between the individual trials. While the average performance of the models was significantly different by subject, certain individual trials failed to show significance. For example, trial one for GPT-3.5 showed significance (p= .164) while trial two failed to show significance (p-value=.023). There was a similar variance between step 1 and step 2 questions and their individual trials.

## Discussion

This study aimed to evaluate the performance of AI language models, namely two versions of ChatGPT, on different USMLE-style medical topics. ChatGPT-4 demonstrated superior performance compared to ChatGPT-3.5, which has been demonstrated in other studies [12,19]. GPT-4’s performance suggests that AI models are improving in their ability to answer complex medical questions. The higher concordance between trials for GPT-4 shows improved consistency and reliability as a medical education tool. Additionally, while GPT-3.5 was inferior to AMBOSS users, GPT-4 is the first ChatGPT model to surpass human performance on USMLE-style AMBOSS tests [17].

While there was variation in performance by medical topic for both models, there was no clear pattern of discrepancy. Notably, both models overall performed best on questions about the male reproductive system. However, this was not consistent between trials or step exam content. This suggests that AI language models do not have certain areas or medical topics of weakness as suggested by poor performance on previous examinations [11]. The variability in AI ability described in the medical literature is perhaps a factor of exam question complexity or AI consistency rather than subject matter. However, the variability may also be explained by limitations in sample size or question selection. Regardless, our results underscore the need for multiple trials when conducting multiple-choice question-based research with ChatGPT.

Both GPT models and AMBOSS users had a linear decline in performance with increased question difficulty, which has been described previously [20]. However, GPT-4's drop in performance was significantly less than that of both AMBOSS users and the GPT-3.5 model. This suggests the model’s greater capacity to handle complex questions than both previous GPT generations and human learners. This capability is a promising finding for medical education.

The criteria for question selection present an important limitation of this study design. Questions that had tables, images, or figures were excluded due to the current limitations in AI's ability to interpret visual content. This exclusion is significant because a substantial amount of USMLE questions incorporate one or more of these three elements. Additionally, there is inherent bias as all questions were sourced from a single-question bank. Therefore, this study cannot fully represent AI performance across the breadth of content on USMLE exams. This limitation highlights the need for future advancement in AI models to handle more complex types of media entries.

## Conclusions

This study demonstrates the improving ability of AI language models, particularly ChatGPT-4, to tackle complex medication questions. Although AI performance varied by medical topic, the lack of a clear pattern suggests its inconsistencies may relate more to question complexity rather than subject matter. ChatGPT-4's reduced decline in performance on more difficult questions highlights its potential to support medical learners with challenging material. Further development and evaluation of AI language models are crucial to integrating AI into medical education.

## Appendices

**Table 2:** Examples of questions used from AMBOSS Qbank Citation: AMBOSS GmbH. QBank. https://amboss.com/. Accessed December 17, 2023 [18].

Category	Question	ChatGPT Response
Behavioral Health, Step 1 Content	A 27-year-old woman comes to the physician because of poor sleep for the past 2 months. She has been gradually sleeping less because of difficulty initiating sleep at night. She does not have trouble maintaining sleep. On average, she sleeps 4–5 hours each night. She feels tired throughout the day but does not take naps. She has attempted relaxation techniques and measures to improve her sleep hygiene without success. The patient does not smoke cigarettes, drink alcohol, or use illicit drugs. Mental status examination shows an anxious mood. The patient asks for a sleeping aid but does not want to feel drowsy in the morning because she has to drive her daughter to kindergarten. Short-term treatment with which of the following drugs is the most appropriate pharmacotherapy for this patient's symptoms? A. Amitriptyline B. Zaleplon C. Trazodone D. Suvorexant E. Phenobarbital	Answer: B. Zaleplon Explanation: Zaleplon is a short-acting non-benzodiazepine hypnotic that helps with sleep initiation and has minimal next-day drowsiness.
Behavioral Health, Step 2 Content	A 23-year-old man is brought to the emergency department by his mother because of a change in his personality. The mother reports that over the past 7 months her son has become increasingly withdrawn; he has not answered any phone calls or visited his family and friends. He had always been a quiet boy, who needed some time on his own, but he used to meet with friends at least once a week. The patient says that he cannot leave the house because aliens are watching him and are “stealing his thoughts”. He does not drink alcohol. He used to smoke cannabis occasionally but quit 1 year ago. His vital signs are within normal limits. Physical examination shows no abnormalities. On mental status examination, it is impossible to follow the patient's sequence of thoughts. He is anxious and has a flat affect. Which of the following is the most likely diagnosis? A. Brief psychotic disorder B. Schizotypal personality disorder C. Delusional disorder D. Mood disorder with psychotic features E. Schizoaffective disorder F. Paranoid personality disorder G. Substance-induced psychosis H. Schizophreniform disorder I. Schizoid personality disorder J. Schizophrenia	Answer: J. Schizophrenia Explanation: The patient exhibits delusions, thought disorder, and negative symptoms lasting over 6 months, characteristic of schizophrenia.
Biostatistics, Epidemiology/Population Health & Interpretation of the Medical Literature, Step 1 Content	The height of American adults is expected to follow a normal distribution, with a typical male adult having an average height of 69 inches with a standard deviation of 0.1 inches. An investigator has been informed about a community in the American Midwest with a history of heavy air and water pollution in which a lower mean height has been reported. The investigator plans to sample 30 male residents to test the claim that heights in this town differ significantly from the national average based on heights assumed to be normally distributed. The significance level is set at 10% and the probability of a type 2 error is assumed to be 15%. Based on this information, which of the following is the most accurate estimate of the statistical power of the proposed study? A. 0.85 B. 0.15 C. 0.10 D. 0.90 E. 0.05	Answer: A. 0.85 Explanation: Statistical power is calculated as 1 minus the probability of a type 2 error (beta), which is given as 0.15.
Biostatistics, Epidemiology/Population Health & Interpretation of the Medical Literature, Step 2 Content	A first-year medical student is conducting a summer project with his medical school's pediatrics department using adolescent intelligence quotient (IQ) data from a database of 1,252 patients. He observes that the mean IQ of the dataset is 100. The standard deviation was calculated to be 10. Assuming that the values are normally distributed, approximately 87% of the measurements will fall in between which of the following limits? A. 70–130 B. 65–135 C. 85–115 D. 80–120 E. 90–110 F. 95–105	Answer: D. 80–120 Explanation: Approximately 87% of a normal distribution falls within ±1.5 standard deviations, which covers the range 80 to 120 for this dataset.
Blood & Lymphoreticular Systems, Step 1 Content	A 46-year-old man is admitted to the hospital with a 3-day history of productive cough with purulent sputum and fever with chills. On the second day of admission, he develops bloody vomiting, altered mental status, and multiple red spots all over the body. He is oriented only to self. His temperature is 39.3°C (102.7°F), pulse is 110/min, respirations are 26/min, and blood pressure is 86/50 mm Hg. Physical examination shows ecchymoses on both lower extremities. Crackles are heard at the right lung base. Laboratory studies show a platelet count of 45,000/mm^3, with a prothrombin time of 44 sec and partial thromboplastin time of 62 sec. D-dimer concentrations are elevated. Which of the following is the most likely cause of this patient's ecchymoses? A. Disseminated intravascular coagulation B. Immune thrombocytopenic purpura C. Severe hepatic dysfunction D. Thrombotic thrombocytopenic purpura E. Hemolytic uremic syndrome	Answer: A. Disseminated intravascular coagulation Explanation: The combination of low platelets, prolonged coagulation times, and elevated D-dimer indicates DIC.
Blood & Lymphoreticular Systems, Step 2 Content	A 53-year-old woman comes to the emergency department because of blurry vision, headache, and multiple episodes of nosebleeds over the last few weeks. She reports that she has also been itching a lot, especially after getting ready for work in the mornings. She has had an 8-kg (17.6-lb) weight loss and increasing fatigue during the past 6 months. Her temperature is 37.8°C (100.0°F), pulse is 80/min, respirations are 15/min, and blood pressure is 158/90 mm Hg. Physical examination shows no lesions or evidence of trauma in the nasal cavity. Her face, palms, nail beds, oral mucosa, and conjunctiva appear red. Abdominal examination shows splenomegaly. Her hemoglobin concentration is 19 g/dL, hematocrit is 58%, platelets are 450,000/μL, and erythropoietin level is below normal. Which of the following is the most likely underlying cause of this patient's condition? A. Stress erythrocytosis B. Mutated JAK2 gene C. Increased intracranial pressure D. Megakaryocyte proliferation E. Erythropoietin receptor mutation F. Renal cell carcinoma	Answer: B. Mutated JAK2 gene Explanation: The symptoms and laboratory findings suggest polycythemia vera, often associated with a JAK2 mutation.
Cardiovascular System, Step 1 Content	A 67-year-old man with stable coronary artery disease comes to the physician for a follow-up examination. Aside from occasional exertional chest pain on mowing the lawn or prolonged jogging, he feels well. He goes jogging for 20 minutes once a week and takes a tablet of sublingual nitroglycerine prior to his run to prevent anginal chest pain. The patient would like to run longer distances and asks the physician whether he could increase the dose of the drug prior to running. Administration of higher dosages of this drug is most likely to result in which of the following? A. Rebound angina B. Development of tolerance C. Reflex sympathetic activity D. Anaphylactic reaction E. Coronary artery vasospasm F. Symptoms of withdrawal	Answer: B. Development of tolerance Explanation: Increasing the dosage of nitroglycerine can lead to tolerance, reducing its effectiveness.
Cardiovascular System, Step 2 Content	A 54-year-old woman comes to the physician because of a 6-month history of dull, persistent pain and swelling of her right leg. The pain is worse at the end of the day and is relieved by walking or elevating her feet. Two years ago, she developed acute deep vein thrombosis in her right calf after a long flight, which was treated with anticoagulants for 6 months. Physical examination shows 2+ pitting edema of her right leg. The skin around the right ankle shows a reddish-brown discoloration and multiple telangiectasias. She has dilated varicose veins in the right leg. Which of the following is most likely to establish the diagnosis? A. Computerized tomography scan with contrast B. Nerve conduction studies C. D-dimer assay D. Ankle-brachial pressure index E. Duplex ultrasonography	Answer: E. Duplex ultrasonography Explanation: Duplex ultrasonography is the preferred method to assess venous insufficiency and varicose veins.
Endocrine System, Step 1 Content	A 42-year-old woman comes to the physician with acute, severe pain in the middle of her lower back. She also complains of constipation and trouble sleeping recently. Menses occur regularly at 28-day intervals. Examination shows localized tenderness to palpation over the lumbar spine. Serum calcium is 14 mg/dL and serum phosphorus is 1.5 mg/dL. An x-ray of the lumbar spine shows a compression fracture of the L4 vertebral body and osteopenia. Which of the following most likely contributes to this patient's decreased bone mineral density? A. Decrease in ovarian estrogen production B. Increase in calcitonin secretion C. Increase in interleukin-1 secretion D. Decrease in alkaline phosphatase secretion E. Decrease in RANKL receptor expression	Answer: C. Increase in interleukin-1 secretion Explanation: Interleukin-1 increases osteoclast activity, contributing to bone resorption and decreased bone density.
Endocrine System, Step 2 Content	A 68-year-old woman who is homeless is brought to the emergency department by ambulance 20 minutes after being found lethargic and confused in front of the grocery store where she usually sleeps. An intramuscular injection of naloxone was administered by paramedics on the way to the emergency department. On arrival, the patient is oriented only to person. Her temperature is 34.8°C (94.6°F), pulse is 49/min, respirations are 9/min, and blood pressure is 83/50 mm Hg. The pupils have a normal diameter and are reactive to light. Deep tendon reflexes are 1+ bilaterally. Physical examination shows periorbital edema, nonpitting edema in both lower extremities, and healed needle track marks in both antecubital fossae. There is no jugular venous distention. Cardiac examination shows a normal S1 and S2. Blood samples are drawn. Serum studies show a sodium concentration of 131 mEq/L, a potassium concentration of 4.9 mEq/L, and a glucose concentration of 54 mg/dL. Arterial blood gas analysis on room air shows: pH= 7.30 PCO2= 52 mm Hg PO2= 70 mm Hg HCO3-= 26 mEq/L An ECG shows bradycardia, low-voltage QRS complexes, and widespread T-wave inversions. The patient is intubated, mechanically ventilated, and covered with a warm blanket. Treatment with warmed intravenous saline, dextrose, thiamine, and epinephrine infusion is initiated. Which of the following is the most appropriate next step in management? A. Hydrocortisone and thyroid hormone replacement therapy B. Emergency hemodialysis C. Emergency pericardiocentesis D. Activated charcoal and sodium bicarbonate therapy E. Flumazenil therapy F. CT scan of the head	Answer: A. Hydrocortisone and thyroid hormone replacement therapy Explanation: The patient's presentation is consistent with myxedema coma, requiring urgent thyroid hormone and cortisol replacement.

## References

[1] Smith TM (2024). How ChatGPT could help medical students learn on the fast track. https://www.ama-assn.org/education/changemeded-initiative/how-chatgpt-could-help-medical-students-learn-fast-track.

[2] Lee H (2024). The rise of ChatGPT: exploring its potential in medical education. Anat Sci Educ.

[3] Tsang R (2023). Practical applications of ChatGPT in undergraduate medical education. J Med Educ Curric Dev.

[4] Zuckerman M, Flood R, Tan RJ, Kelp N, Ecker DJ, Menke J, Lockspeiser T (2023). ChatGPT for assessment writing. Med Teach.

[5] Cheung BH, Lau GK, Wong GT (2023). ChatGPT versus human in generating medical graduate exam multiple choice questions-a multinational prospective study (Hong Kong S.A.R., Singapore, Ireland, and the United Kingdom). PLoS One.

[6] Kung TH, Cheatham M, Medenilla A (2023). Performance of ChatGPT on USMLE: potential for AI-assisted medical education using large language models. PLOS Digit Health.

[7] Guerra GA, Hofmann H, Sobhani S (2023). GPT-4 artificial intelligence model outperforms ChatGPT, medical students, and neurosurgery residents on neurosurgery written board-like questions. World Neurosurg.

[8] Terwilliger E, Bcharah G, Bcharah H, Bcharah E, Richardson C, Scheffler P (2024). Advancing medical education: performance of generative artificial intelligence models on otolaryngology board preparation questions with image analysis insights. Cureus.

[9] Moshirfar M, Altaf AW, Stoakes IM, Tuttle JJ, Hoopes PC (2023). Artificial intelligence in ophthalmology: a comparative analysis of GPT-3.5, GPT-4, and human expertise in answering StatPearls questions. Cureus.

[10] Haddad F, Saade JS (2024). Performance of ChatGPT on ophthalmology-related questions across various examination levels: observational study. JMIR Med Educ.

[11] Suchman K, Garg S, Trindade AJ (2023). Chat Generative pretrained transformer fails the multiple-choice American College of Gastroenterology self-assessment test. Am J Gastroenterol.

[12] Massey PA, Montgomery C, Zhang AS (2023). Comparison of ChatGPT-3.5, ChatGPT-4, and orthopaedic resident performance on orthopaedic assessment examinations. J Am Acad Orthop Surg.

[13] Ariyaratne S, Jenko N, Mark Davies A, Iyengar KP, Botchu R (2024). Could ChatGPT pass the UK radiology fellowship examinations?. Acad Radiol.

[14] Knoedler L, Alfertshofer M, Knoedler S (2024). Pure wisdom or Potemkin villages? A comparison of ChatGPT 3.5 and ChatGPT 4 on USMLE Step 3 style questions: quantitative analysis. JMIR Med Educ.

[15] Danehy T, Hecht J, Kentis S, Schechter CB, Jariwala SP (2024). ChatGPT performs worse on USMLE-style ethics questions compared to medical knowledge questions. Appl Clin Inform.

[16] Brin D, Sorin V, Vaid A (2023). Comparing ChatGPT and GPT-4 performance in USMLE soft skill assessments. Sci Rep.

[17] Gilson A, Safranek CW, Huang T, Socrates V, Chi L, Taylor RA, Chartash D (2023). How does ChatGPT perform on the United States Medical Licensing Examination (USMLE)? The implications of large language models for medical education and knowledge assessment. JMIR Med Educ.

[18] (2024). AMBOSS GmbH. AMBOSS Qbank. http://amboss.com/.

[19] Ali R, Tang OY, Connolly ID (2023). Performance of ChatGPT, GPT-4, and Google Bard on a neurosurgery oral boards preparation question bank. Neurosurgery.

[20] Knoedler L, Knoedler S, Hoch CC (2024). In-depth analysis of ChatGPT's performance based on specific signaling words and phrases in the question stem of 2377 USMLE step 1 style questions. Sci Rep.

